# The Potential Role of Senescence As a Modulator of Platelets and Tumorigenesis

**DOI:** 10.3389/fonc.2017.00188

**Published:** 2017-08-28

**Authors:** Claudio A. Valenzuela, Ricardo Quintanilla, Rodrigo Moore-Carrasco, Nelson E. Brown

**Affiliations:** ^1^Center for Medical Research, University of Talca Medical School, Talca, Chile; ^2^Faculty of Health Sciences, University of Talca, Talca, Chile

**Keywords:** cancer, fibrinolysis, platelets, senescence, thrombosis

## Abstract

In addition to thrombus formation, alterations in platelet function are frequently observed in cancer patients. Importantly, both thrombus and tumor formation are influenced by age, although the mechanisms through which physiological aging modulates these processes remain poorly understood. In this context, the potential effects of senescent cells on platelet function represent pathophysiological mechanisms that deserve further exploration. Cellular senescence has traditionally been viewed as a barrier to tumorigenesis. However, far from being passive bystanders, senescent cells are metabolically active and able to secrete a variety of soluble and insoluble factors. This feature, known as the senescence-associated secretory phenotype (SASP), may provide senescent cells with the capacity to modify the tissue environment and, paradoxically, promote proliferation and neoplastic transformation of neighboring cells. In fact, the SASP-dependent ability of senescent cells to enhance tumorigenesis has been confirmed in cellular systems involving epithelial cells and fibroblasts, leaving open the question as to whether similar interactions can be extended to other cellular contexts. In this review, we discuss the diverse functions of platelets in tumorigenesis and suggest the possibility that senescent cells might also influence tumorigenesis through their ability to modulate the functional status of platelets through the SASP.

## Introduction

Platelets are key blood components that are continuously generated in the bone marrow through fragmentation of the edges of megakaryocytes (1). In addition to their canonical role in hemostasis, platelets participate in a variety of pathological processes, including chronic inflammation and cancer (2). As the incidence of both cancer and chronic inflammatory disorders rise in an age-dependent manner, the influence that aging may exert on platelet function has become particularly relevant (3). So far, however, the mechanisms involved in this age-dependent modulation of platelets or other components of the hemostasis cascade remain poorly characterized. Similarly, the age-dependent factors that modulate the interaction between platelets and cancer cells are largely unknown. Based on the capacity of senescent cells to actively modify the tissue microenvironment through the secretion of pro-inflammatory mediators, herein we speculate about the existence of a functional link between cellular senescence and platelets that may help explain the increased incidence of cancer and thrombotic diseases in older individuals.

## The Complex Involvement of Platelets in Cancer

**Figure 1:**
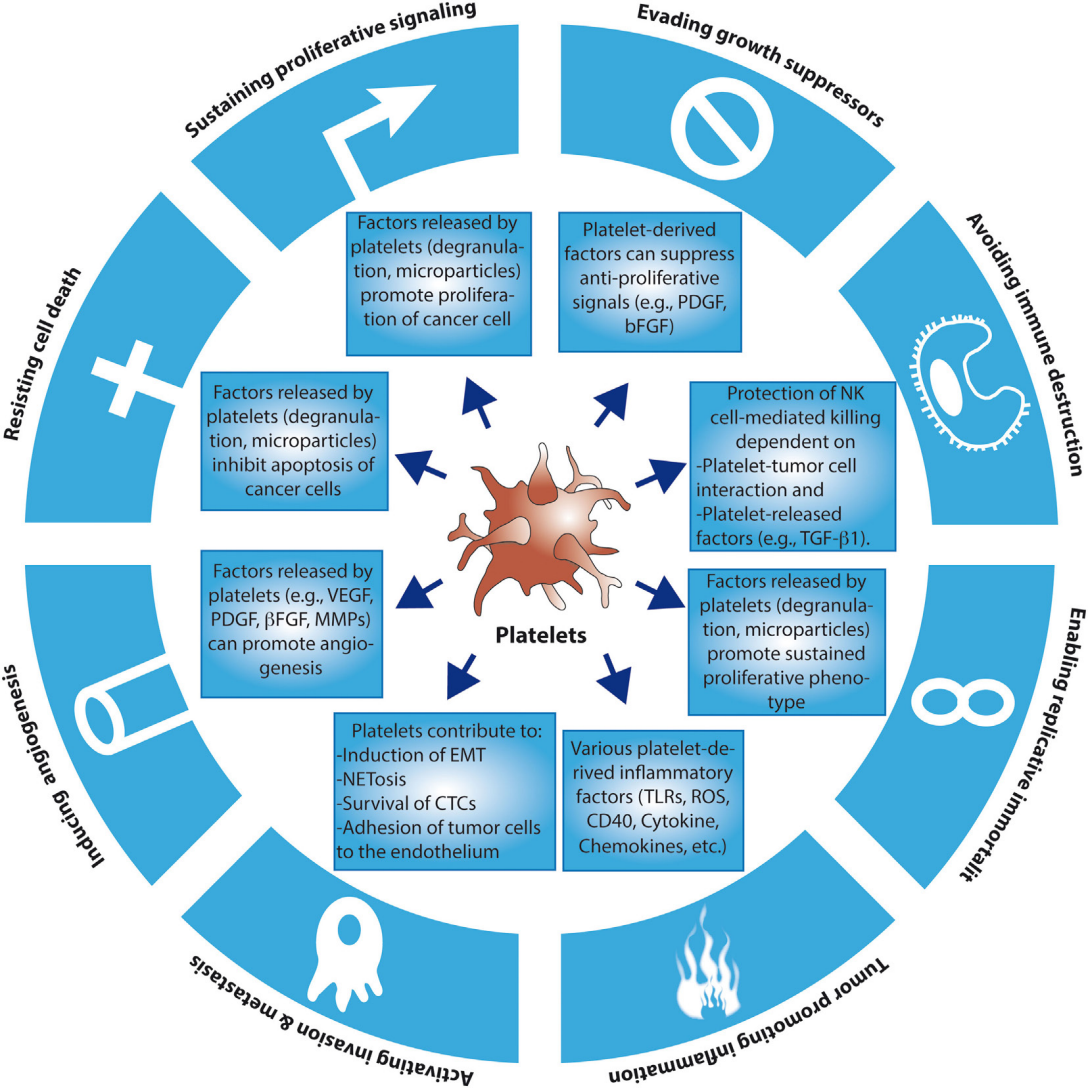
The contributions of platelets to the hallmarks of cancer. So far, there is evidence that platelets may contribute to the emergence of several of the hallmarks of cancer through the release of soluble factors and microparticles, or through direct cancer cell–platelet interactions. For simplicity reasons, more complex and intricate mechanisms have been excluded. EMT, epithelial–mesenchymal transition; CTCs, circulating tumor cells; TLRs, toll-like receptors; ROS, reactive oxygen species; NKs, natural killers.

The functional connection between cancer and platelets has been recognized since the late nineteenth century, when an association between the occurrence of certain solid tumors and the development of venous thrombosis and blood hypercoagulability was first described (4). Accordingly, defects in platelet function or reduced platelet counts have both been associated with a reduced ability of tumors to metastasize (5, 6). We now know that platelets may contribute to the establishment of various hallmarks of cancer, including the ability of cancer cells to sustain proliferation, to resist apoptosis and to promote angiogenesis and metastasis (1) (for an overview of the contribution of platelets to the hallmarks of cancer, see Figure 1). It is presently unclear, however, to what extent these contributions are the result of a direct action of platelets on tumor cells or, alternatively, may be part of an underlying inflammatory process inherent to many tumors. Inflammatory cells and soluble mediators of inflammation are important constituents of the tumor microenvironment. In some tumors, inflammatory conditions are present before the occurrence of malignant transformation (7). Yet in other types of tumors, the inflammatory microenvironment emerges during the process of neoplastic transformation (8). Regardless of its origin, an environment rich in inflammatory cells and growth factors is thought to promote proliferation, angiogenesis, and/or metastasis of cancer cells (1, 7).

Platelets participate in diverse inflammatory processes that may be associated with cancer (9, 10). One of the crucial inflammatory mechanisms involving platelets is NETosis. In this process, neutrophils release part of their intracellular content (chromatin, histones, enzymes, etc.) to the extracellular milieu. These components can then form a mesh that captures circulating microbes and impedes their tissue adhesion and colonization (11, 12). Mechanistically, granulocyte colony-stimulating factor (CSF-G) released by tumor cells is thought to increase the production of inflammatory neutrophils and promote neutrophil–platelet interaction (*via* P-selectin), which in turn is required to stimulate NETosis and a hyper-coagulation/pro-thrombotic state (13). More recently, NETosis has also been shown to play a role at different stages of tumorigenesis, including metastasis (14, 15), and the establishment of paraneoplastic syndromes leading to organ failure and thrombosis (16). Other components of innate immunity that have been associated with cancer are the inflammatory responses mediated by toll-like receptors (TLRs). Classic mediators of TLR activation are tissue damage-associated proteins, particularly members of the HMGB1 (high-mobility group box1). Whereas under normal conditions these proteins are bound to chromatin, they can be released by necrotic cells or secreted by macrophages under inflammatory or tissue damaging conditions (17). Importantly, Le-Xing et al. demonstrated that toll-like receptor 4 (TLR4), present in platelets and other cells of myeloid origin, is crucial for the interaction between tumor cells and platelets (18). Taken together, these examples illustrate the importance of platelets in the regulation of diverse pro-tumorigenic inflammatory processes.

In addition to the general roles of platelets in inflammation, activated platelets may also participate more directly in tumor growth and metastasis. The alpha granules of platelets are the source of various trophic factors, including growth factors, chemokines, adhesion molecules, and angiogenic factors, which may promote tumor progression once they are released by activated platelets (19). In fact, the levels of many of these factors have been used as prognostic determinants in cancer patients (20, 21). In addition to these paracrine actions, tumor growth and metastasis also seem to depend on the ability of platelets to physically interact with tumor cells through specific integrin complexes. For example, blockade of GpIIb/IIIa—a fibrinogen-binding integrin complex that is required for platelet aggregation and binding to tumor cells—reduces the number of metastatic nodules in the lung (22). Consistent with this observation, mice deficient in β3-integrin also display reduced metastasis (6). Altogether, these data indicate that integrin-mediated tumor cell–platelet interaction is necessary for platelet activation during metastasis (23). As mentioned above, TLR4 can also enhance tumor cell–platelet interactions, a function that is, at least in part, dependent on the release of endogenous ligand HMGB1 by tumor or damaged cells (18).

The growth factor-enriched microenvironment generated by platelet degranulation can also render tumor cells more resistant to chemotherapeutic agents (Figure 1). For example, in a group of patients with recurrent ovarian cancer, an increased number of platelets were associated with a reduction in overall survival and resistance to chemotherapy (24, 25). A similar phenomenon occurs in gastric cancer, where increases in both the number and volume of platelets were associated with a reduced response to chemotherapy (26, 27). Platelets also increase the overall survival of 5-fluorouracil- and paclitaxel-treated colon adenocarcinoma cells (28). In this case, the presence of platelets induces the expression of anti-apoptotic proteins and reduces the expression of pro-apoptotic proteins in cancer cells (28). This prosurvival effect seems to correlate with the ability of platelets to change the profile of factors secreted by cancer cells themselves, which may explain the reduced apoptotic effect of 5-fluorouracil y paclitaxel (28). Interestingly, the anti-tumorigenic effects of thrombocytopenia may also be explained by the presence of micro-hemorrhages that improve chemotherapy response (29–32). In addition to influencing anti-cancer therapy response, platelet function itself can be altered in the course of chemotherapy. For example, Kedzierska et al. described hematological alterations in patients with breast cancer before, during and after chemotherapy (33), demonstrating that the size, number, and aggregation capacities of platelets obtained from patients undergoing chemotherapy were higher compared to healthy controls (33). These changes appear to be a compensatory mechanism that hinders the correct distribution of the chemotherapeutic drugs within the tumor. Recently, Holmes et al. (34) also described changes in the secretory profile of platelets in patients with breast cancer. They observed a differential regulation in the release of angiogenic factors, especially vascular endothelial growth factor (VEGF), by platelets from individuals with cancer versus healthy individuals. Interestingly, these authors also showed that platelets from individuals undergoing chemotherapy released more angiogenic factors compared to individuals with cancer but not subjected to chemotherapy treatment (34).

Platelets may also promote distant colony formation (metastasis) by allowing the survival of tumor cells in the circulation [circulating tumor cells (CTCs)] (35). Under normal conditions, CTCs are rapidly eliminated from circulation by the host immune system or the activation of apoptosis (following lack of substrate attachment, a form of apoptosis known as anoikis). However, CTCs that become coated with platelets are protected from immune-dependent cell lysis (36). In this scenario, adhesion molecules present on the surface of activated platelets, including GpIIb/IIIa integrin, mediate the formation of heteroaggregates with tumor cells that remain shielded from immunological detection and natural killer (NK) cell-mediated lysis (35, 37). At least in part, this immunological tolerance may be also explained by platelet-derived secreted factors, such as TGF-β1, that impair NK cell anti-tumor activity (38).

Platelets may also facilitate the adhesion of tumor cells to the endothelium, generating a locally protected tumor microenvironment that promotes migration of tumor cells. The establishment of this microenvironment also seems depend on granulocyte recruitment (39) and a platelet-induced increase in endothelial permeability (40). It has been shown that this effect depends on the ability of activated platelets to secrete nucleotides that act on P2Y2 receptors expressed on the surface of endothelial cells. It is important to mention that platelets are also considered a major source of VEGF, an angiogenic factor that is released upon activation (41). In addition, platelet-derived TGF-β1 enables tumor cells to undergo a process that resembles the epithelial–mesenchymal transition, thus facilitating invasion and dissemination (42). Finally, platelet-derived microparticles also play a role in tumor growth, migration, and metastasis (43). CTCs can increase the production of platelet-associated microparticles that promote invasiveness and metastasis (44). Among other proteins, microparticles also contain tissue factor, which is important for the generation of thrombin and the subsequent activation of protease-activated receptor-1 receptors on platelets, leading to VEGF secretion and angiogenesis (45). Taken together, the role of platelets in tumor invasion and metastasis is complex and can be explained by both direct actions on cancer cells or through collaborative effects with other cell types. So far, platelet-assisted dissemination of cancer cells has been demonstrated in the context of several human cancers, including colorectal (46), lung (47), breast (48), kidney (49), and pancreatic (50) cancers.

Tumor-derived factors leading to platelet production and activation are similarly variable and, in general, poorly understood. Several pro-inflammatory cytokines released by tumor cells, or tumor-associated stromal cells, are able to increase the number of platelets by stimulating the formation and fragmentation of megakaryocytes (51). Among the most recent findings, Stone et al. (24) reported that thrombocytosis in patients with ovarian cancer was associated with cytokine production by tumor and host tissues. In particular, tumor-derived interleukin-6 (IL-6) led to an increase in the number of activated platelets (24). Similarly, local secretion of soluble mediators by tumor cells enhances platelet activation and aggregation. For example, colorectal cancer cells induce platelet aggregation *via* the release of ADP and MMP-2 (52). Platelet aggregation, in turn, correlated with overexpression of GPIIb/IIIa and P-selectin in platelets, allowing the formation of tumor cell–platelet interactions (5, 52). Some of these mechanisms also involve the generation of thrombin (e.g., colon carcinoma cells) (53). Other mechanisms of cancer-dependent platelet activation that involve cell-to-cell contact include the overexpression of podoplanin, a trans-membrane protein (also known as “aggrus”) that is expressed in several tumor types (54). Podoplanin binds the c-type lectin receptor on the surface of platelets, triggering their activation (55, 56). Similarly, the release of cathepsin B by B16 melanoma cells can also trigger the activation of platelets (57).

## Aging and Platelets

So far, the role of the physiological process of aging as a modulator of platelet function, or as a factor that may influence the interaction between platelets and tumor cells, remains poorly understood (58). Early studies found that plasma concentrations and activities of various coagulation factors (fibrinogen, von Willebrand factor, factors V, VII, VIII, and IX) increase with the physiological process of aging (3, 59, 60). Among these factors, fibrinogen is particularly relevant because it represents a primary risk factor for thrombotic disorders (61, 62). Interestingly, fibrinogen levels also increase in response to the pro-inflammatory cytokine IL-6. As levels of IL-6 were also strongly correlated with aging (63), these findings might suggest that high levels of fibrinogen in the elderly could be, at least in part, a reflection of an age-dependent inflammatory state. Similarly, the fibrinolytic system is also affected by aging. Thus, several studies have shown that the levels of PAI-1 (plasminogen activator inhibitor-1), a major inhibitor of fibrinolysis, increase with age (3, 64).

In addition to the above-mentioned hemostatic factors, platelets and endothelial cells are also affected by aging. Decrease in bleeding time (a surrogate for platelet activity) and elevation of markers of platelet activation have both been correlated with physiological aging (65). Moreover, platelets from older individuals display a greater aggregation response to ADP and collagen compared to younger individuals (66). Similarly, endothelial cells isolated from older individuals display important changes that may predispose these individuals to thrombotic disease. These changes include an age-dependent decline in endothelial production of prostacyclin and nitric oxide (67, 68).

Taken together, changes in virtually all aspects of hemostasis have been associated with physiological aging. As older adults often show signs of chronic inflammation, it is likely that changes in hemostasis—particularly those involving platelet function—may be part of a more general inflammatory process. So far, however, the age-dependent mechanisms involved in the modulation of hemostasis and platelet function are not completely understood. In the next sections, we advance the idea that cellular senescence might explain, at least in part, some of the hemostatic changes that lead to thrombosis and cancer.

## Cellular Senescence

Typical hallmarks of physiological aging include impaired tissue regeneration and repair, a functional impairment of progenitor cells, and alterations of the immune system (69). While the specific cellular changes associated with each one of these hallmarks will vary depending on the tissue analyzed, cellular senescence is rapidly emerging as an underlying process that may help explain some of these changes. In keeping with this idea, senescent cells accumulate in several tissues derived from aged animals (70, 71).

Cellular senescence was described more than 50 years ago as a process that limits the proliferation of primary human cells propagated *in vitro* (72). Simply stated, cellular senescence refers to a type of permanent and stable cell cycle arrest induced by numerous stimuli, including DNA damage, oxidative stress, activation of certain oncogenes, and therapeutic stress (including chemotherapy and radiotherapy). Because senescent cells cease to proliferate, cellular senescence was initially regarded as a functional equivalent of apoptosis in its ability to suppress tumor formation (73). However, recent work indicates that the physiological relevance of cellular senescence extends far beyond tumor suppression, into processes as diverse as embryonic development, wound healing, and tissue repair (74–77). Moreover, as discussed in the next section, the presence of senescent cells in tissues may actually promote the acquisition of neoplastic features by adjacent cells or otherwise foster the generation of a pro-inflammatory environment (78).

**Figure 2:**
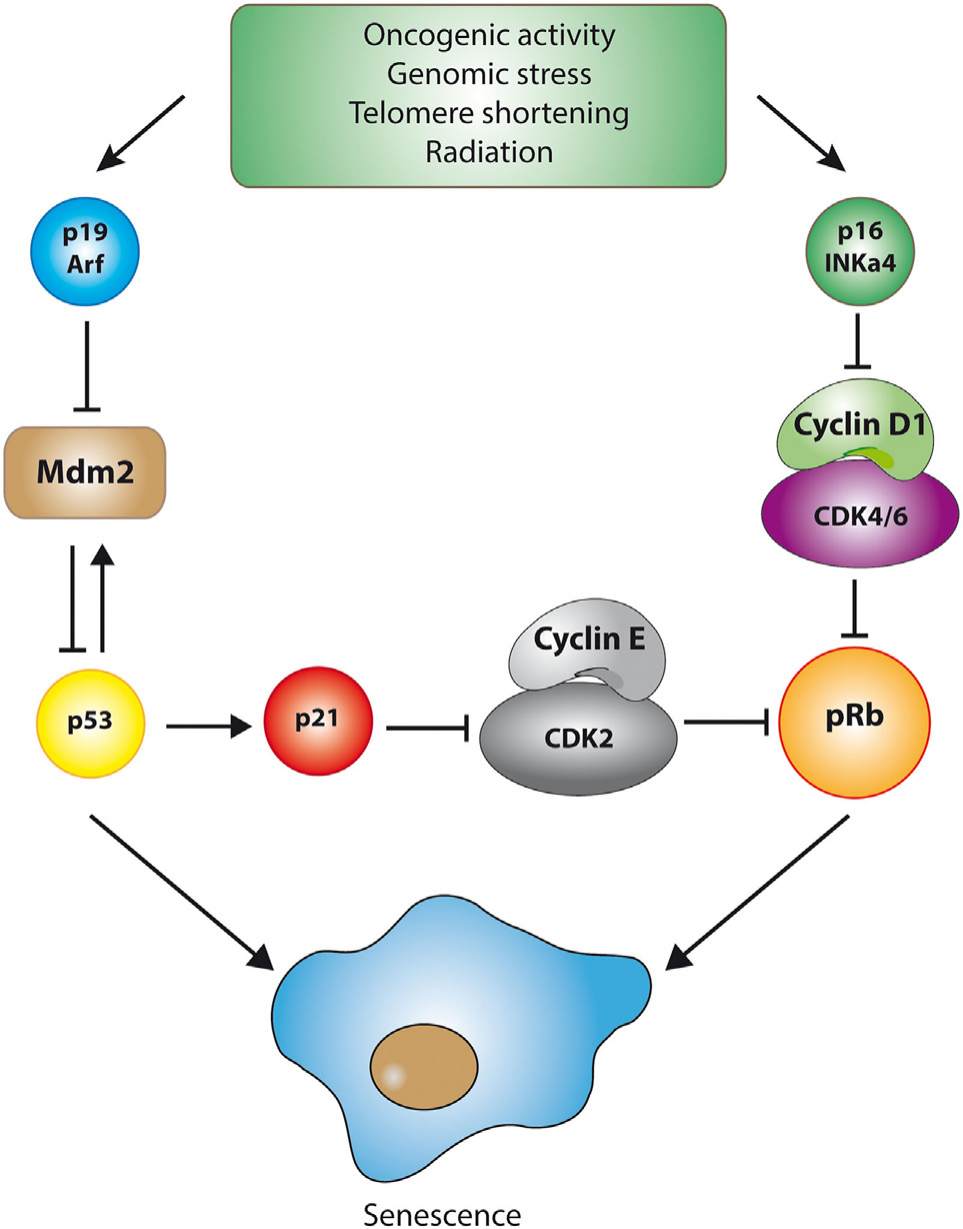
The p53 and pRB pathways during senescence. Cellular senescence is triggered by the activation of one of two major tumor suppressive pathways, namely the p53 or the pRB pathway. Although the details may vary, activation of these pathways is marked by the inhibition of cyclin-dependent kinases (CDKs), the key enzymes involved in cell cycle progression. For example, stabilization of the transcription factor p53 in response to DNA damage (which is dependent on p19/ARF-mediated inhibition of MDM2, an E3-ubiquitin ligase that targets p53 for degradation) is followed by the p53-dependent transcription of genes involved in the orchestration of cellular senescence. One of these target genes, p21^Cip1/WAF1^, is a potent inhibitor of cyclin/CDK complexes, particularly cyclin E/CDK2 complexes. Similarly, p16^INK4a^, another inhibitor of CDKs that highly expressed in some senescent cells, inhibits cyclin D1/CDK4/6 complexes. The upregulation of both types of CDK inhibitors results in the inhibition of CDKs and the subsequent activation of the pRB pathway, which effectively blocks the G1-S cell cycle transition.

Morphologically, senescent cells appear large and “flattened” (79) and are typically positive for β-galactosidase activity at pH 6.0, a reflection of the high content of lysosomes in these cells (78, 80). Another prominent feature of senescent cells is the presence of “senescence-associated heterochromatin foci,” which correspond to regions of chromatin condensation (heterochromatin) that appear as bright and dense foci in the nuclei of senescent cells (81, 82). The cell cycle exit observed in senescent cells is generally associated with a typical DNA content of G1 phase, that is, a failure to initiate DNA replication even when growth conditions are adequate. The initial transition from cycling to cell cycle arrest involves a reduction in the activity of cyclin/cyclin-dependent kinases (CDKs) complexes, leading ultimately to the activation of the p53 and pRB tumor suppressor pathways (83, 84). For example, the transcription factor p53 can be stabilized in response to various stressful stimuli, increasing the expression of various target genes that trigger cellular senescence or, in extreme cases, apoptosis. One of these targets, p21^Cip1/WAF1^, is a potent inhibitor of cyclin/CDK complexes. Similarly, p16^INK4a^, another inhibitor of CDKs, is highly expressed in senescent cells (83, 85). The upregulation of both types of CDK inhibitors results in the inhibition of CDKs and the subsequent activation (through hypo-phosphorylation) of the pRB pathway, event that effectively blocks the G1-S transition (86) (see Figure 2).

Historically, the extent to which cellular senescence contributes to organismal aging and age-driven tissue dysfunction has been difficult to establish, in part due to the lack of markers that could specifically detect senescent cells in aging tissues (87). Nonetheless, the use of combinations of markers has provided convincing evidence that senescent cells do accumulate in aged tissues, as well as in sites of tissue injury and repair (71, 74, 88). For example, markers of DNA damage and de-repression of the *INK4/ARF* locus—which encodes for the tumor suppressive proteins p16^INK4A^ and p19^ARF^—increase with chronological aging. Accordingly, the levels of p16^INK4A^ correlate with the aging of numerous tissues from mice and humans (89, 90). Moreover, for at least some tissues (e.g., liver, skin, lung, and spleen), a good correlation between the proportion of cells with DNA damage, and the proportion of cells displaying senescence-associated β-galactosidase activity, has been found (71).

Taken together, the current evidence indicates that in addition to functioning as a barrier against tumor formation, cellular senescence is also active during embryonic development, tissue repair, and organismal aging. The involvement of cellular senescence in these physiological processes is currently thought to depend on the ability of senescent cells to produce and secrete a variety of factors that can impinge on neighboring cells and the extracellular matrix (ECM), a function that only becomes evident in the context of complex tissues. As mentioned in the following sections, these non-cell autonomous capabilities of senescent cells are also emerging as key contributors to the pathogenesis of age-related conditions, including chronic inflammation, fibrosis, and, paradoxically, cancer.

## The Senescence-Associated Secretory Phenotype (SASP)

In addition to cell cycle arrest, the establishment of a mature senescent phenotype involves extensive metabolic reprograming, as well as the implementation of complex traits such as the SASP (91, 92). The SASP refers to the almost universal capacity of senescent cells to produce and secrete a variety of soluble and insoluble factors, including extracellular proteases, cytokines, chemokines, and growth factors. This ability of senescent cells to potentially modify the tissue microenvironment (neighboring cells and the ECM) *via* SASP adds a further layer of complexity to the implications of cellular senescence to tissue homeostasis and disease (93–96).

A common feature of aging and age-related diseases is chronic inflammation. The term “inflamm-aging” has been coined to describe a low-grade, chronic, and systemic inflammation associated with aging and aging phenotypes in the absence of evidence of infection (97). In line with this concept, many of the factors secreted by senescent cells are also well-known pro-inflammatory molecules with the potential to induce chronic inflammation in certain biological contexts (69, 98). Indeed, early microarray analyses revealed that senescent fibroblasts display an expression profile that resembles the one displayed by fibroblasts in early stages of wound repair (99). More recently, a unique type of inflammation triggered by senescent cells, the senescence-inflammatory response, has been identified (100). Interestingly, similar to chronic inflammation produced by other mechanisms, the inflammatory “secretoma” produced by senescent cells also seems to depend on activation of the NF-κB and C/EBP-β transcriptional regulators (101). Examples of conserved components of the SASP with known pro-inflammatory actions include IL-6 (102), IL-1-α (103) macrophage inflammatory protein, various metalloproteinases (MMP-2, -4, -1), GM-CSF, and cathepsin B (93, 104).

As expected, the SASP can have complex effects on tissue microenvironments. Thus, some components of the SASP can propagate or reinforce the senescent phenotype through autocrine or paracrine mechanisms, leading to further secretion and amplification of the SASP (105). In addition, SASP factors may attract immune cells, which in turn can orchestrate the elimination of senescent cells and the termination of a senescence-associated inflammatory response. Importantly, clearance of senescent cells seems to dictate the net effect of cellular senescence at the organismal level (106). While transient and limited cellular senescence can be beneficial in the context of the normal tissue remodeling that occurs during embryonic development and wound healing, chronic accumulation of senescent cells—owing to age-dependent deterioration of the innate or adaptive immunity—can have important detrimental consequences. For example, pro-inflammatory cytokines secreted by senescent cells may promote chronic inflammation and, depending on the biological context, lead to pathological conditions characterized by an excess of fibrosis (e.g., liver cirrhosis) (74, 107). Moreover, the SASP, particularly its inflammatory component, can accelerate tumor initiation and progression by fostering a pro-tumorigenic microenvironment (106, 108). Accordingly, clearance of tumor cells (or cells of the tumor stroma) undergoing genetically or drug-induced senescence leads to long-term regression and reduced recurrence of tumors in mouse models of liver and breast tumorigenesis (107, 109–113).

The complex heterotypic interactions in which senescent cells can participate were anticipated by early *in vitro* experiments showing that senescent fibroblasts can enhance proliferation and tumorigenesis of epithelial cells of various types (114–117). For example, factors secreted by senescent fibroblasts, such as amphiregulin and GROα, stimulate the proliferation of premalignant prostate epithelial cells (93, 114). Similarly, high levels of IL-6 and IL-8, also produced by senescent fibroblasts, can promote invasion of weakly malignant keratinocytes (118). Importantly, coinjection of senescent fibroblasts with either premalignant or malignant mammary epithelial cells can lead to, or accelerate, tumor formation in mice (116). Furthermore, normal human prostate epithelial cells undergoing senescence can also enhance *in vivo* tumorigenicity of low- or non-tumorigenic prostate cancer cells, suggesting that factors released by senescent epithelial cells can also be protumorigenic (119). It is worth mentioning that the SASP-dependent ability of senescent cells to promote tumorigenesis has been mainly reported in cellular systems involving co-cultures of epithelial cells and fibroblasts. Therefore, it remains unknown if similar interactions can be observed in other cellular contexts. Finally, it is important to emphasize that not all components of a SASP are pro-tumorigenic. Some SASP components have anti-angiogenic effects or are even able to induce apoptosis or senescence in non-senescent neighboring cells (120, 121).

## The Potential Role of the SASP in Hemostasis

Based on the emerging physiological and pathological processes in which the SASP might be involved, it is conceivable that senescent cells may also affect hemostasis through mechanisms that include, but are not limited to, changes in the production and functional status of platelets. As mentioned elsewhere in this review, IL-6 is one of the most prominent pro-inflammatory cytokines present in the SASP (102). Interestingly, IL-6 has been postulated as a central mediator of age-associated inflammatory pathways (63), with serum concentrations of IL-6 increasing with age (122). Moreover, IL-6 upregulates the synthesis of hemostatic factors, such as fibrinogen, and may also directly activate platelets (63, 123). Thus, it is tempting to speculate that the high levels of IL-6 (and other pro-inflammatory factors, such as IL-1β and TNF-α) detected in aged individuals could reflect, at least in part, an increased rate of secretion of this cytokine by senescent cells—or by other cells responding to senescent cells—in the context of a senescence-induced chronic inflammation. An age-dependent increase of pro-inflammatory factors would, in turn, contribute to platelet activation and a higher proclivity to thrombus formation. Therefore, we postulate that cellular senescence (as a result of physiological aging or secondary to therapeutic stress) might play an important role in the regulation of platelet function. By regulating the activation of platelets, senescent cells could provide yet another mechanism contributing to the higher prevalence of chronic inflammation (and cancer) in aged individuals.

While direct interaction between senescent cells and platelets remains to be experimentally confirmed, components of the SASP have already been linked to the modulation of the process of fibrinolysis *via* the plasminogen activation pathway (93, 124). Thus, increased plasma levels of PAI-1 (plasminogen activator inhibitor-1) are associated with a variety of age-associated conditions, including thrombogenic endothelial dysfunction (93). Supporting the connection between cellular senescence and thrombogenesis, PAI-1 mRNA and protein levels are also constitutively upregulated in senescent endothelial cells (125). In addition, fibroblasts and endothelial cells isolated from elderly donors or from patients with Werner syndrome—a disease characterized by premature aging and atherosclerosis—also display elevated levels of PAI-1 (126). Taking together, these data support the existence of a close association between aging, cellular senescence, and the deterioration of the fibrinolytic system.

Finally, senescent cells also secrete insoluble proteins that are normally present in the ECM and accumulate as a consequence of chronic inflammatory processes. One prominent example is fibronectin, a component of the connective tissue that is also found on cell surfaces, plasma, and other body fluids. Importantly, it has been demonstrated that fibronectin stabilizes the hemostatic clot, controls the diameter of the fibrin fiber, and also enhances platelet adhesion (127).

Taken together, the data support a model in which SASP components could modulate various aspects of hemostasis, including the functional status of platelets. Local activation of platelets, in turn, could contribute propitiate chronic inflammation, accelerate tumor progression, and enhance thrombus formation. A selection of senescence-associated secreted factors that could modify the function or production of platelets is listed in Table 1.

**Table 1:** Senescence-associated secretory phenotype (SASP) factors with potential effect on platelets aggregation and the fibrinolytic system.

SASP component	Function
Interleukin-6	Upregulates the production of hepatic thrombopoetin, elevating the number of platelets number (24)
IL-11	Contributes to megakaryopoiesis and thus indirectly to thrombopoiesis (51, 128)
PAI-1	Main inhibitor of tissue plasminogen activator and urokinase (24), regulates the dissolution of fibrin and also inhibits the degradation of the extracellular matrix by reducing plasmin generation (129)
MMP-2	Released by tumor cells and activated platelets *in vitro* (130)
GM-CSF	Contributes to megakaryopoiesis and thus indirectly to thrombopoiesis (51)
Fibronectin	Involved in cell adhesion and migration processes, including embryogenesis, wound healing, blood coagulation, host defense, and metastasis (131)
THPO	Necessary for megakaryocyte proliferation and maturation, as well as for thrombopoiesis (132)
Granulocyte colony-stimulating factor (G-CSF)	Cancer cell releases high levels of G-CSF primed neutrophils to release NETs, activating platelets (133), and also contributes to megakaryopoiesis and thus indirectly to thrombopoiesis (51)
MMP1	Activates protease-activated receptor-1 (PAR-1) by cleaving the receptor and promotes platelet aggregation through PAR-1 (134)

## Concluding Remarks

The functional interaction between cancer cells and platelets has been well established. Most of the efforts aimed to clarify these interactions have been focused on the ability of tumor cells (or tumor-associated stromal cells) to produce and secrete pro-inflammatory factors that can result in the activation of platelets. Active platelets—acting synergistically with other components of the tumor stroma—can then promote or enhance tumor progression and metastasis. Paradoxically, many of the factors secreted by tumor cells or tumor-associated inflammatory cells with a known effect on platelet activity are also produced and secreted by cells undergoing senescence, a process originally regarded as tumor suppressive. Indeed, the evidence indicates that cellular senescence may also play an active role in driving, rather than suppressing, tumor formation, a non-cell autonomous role that seems to be largely dependent on the SASP. Accordingly, factors released by senescent cells may help create a pro-tumorigenic microenvironment that enhances proliferation and migration of neighbor cells (135). Although still controversial, this model would be in line with the observation that the prevalence of most cancers increases with age.

**Figure 3:**
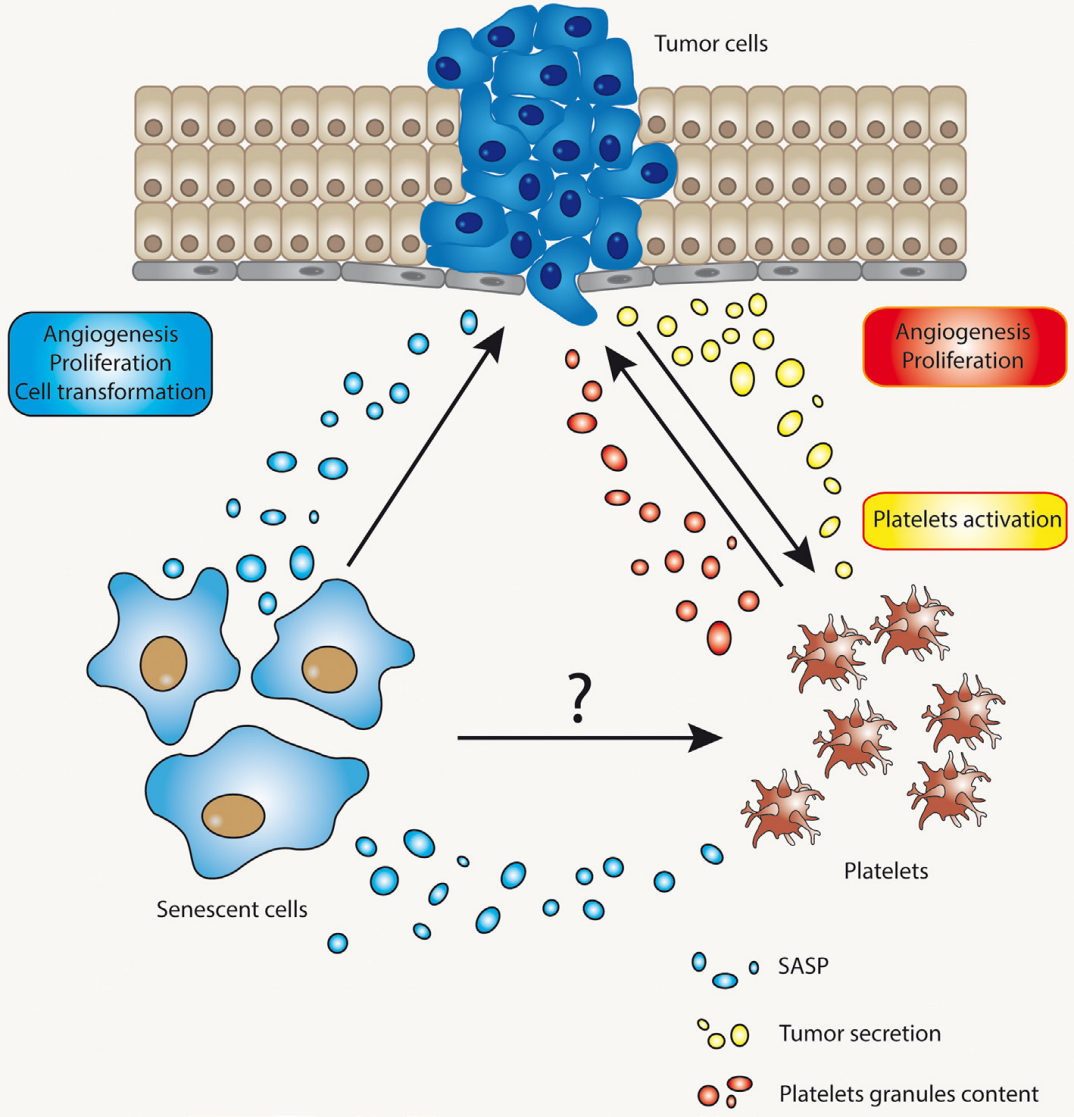
The complex interaction between senescent cells, tumor cells, and platelets. The interaction between tumor cells and platelets is already well known. Tumor cells may affect platelet activation through several mechanisms and, reciprocally, activated platelets may release factors that impinge on proliferation and metastasis of tumor cells, or cells in the process of becoming tumorigenic. Senescent cells, on the other hand, might cause alterations in microenvironment through their ability to develop a secretory phenotype [senescence-associated secretory phenotype (SASP)]. SASP’s components, for example, could alter the functional status of platelets or the process of fibrinolysis.

Alterations in hemostasis involving platelet dysfunction or alterations in the process fibrinolysis are at the core of thrombogenesis (136). As with cancer, thrombogenesis is most commonly observed in older individuals, who presumably harbor a higher proportion of senescent cells in their tissues. We, therefore, postulate that cellular senescence, either as a result of normal aging or secondary to stress, could play an important role in the regulation of platelet function. Figure 3 depicts the potential relationship between senescent cells, platelets, and cells at risk of becoming tumorigenic. According to this model, senescent cells have the ability to modify the microenvironment in ways that may enhance tumorigenesis. Similarly, senescent cells might also regulate the activity of platelets, the process of fibrinolysis, or both. By regulating the activation of platelets, senescent cells may provide yet another mechanism to enhance tumorigenesis. Whether or not these circuits are relevant to tumorigenesis and/or thrombogenesis remains to be fully elucidated.

## Author Contributions

CV contributed to writing the manuscript, figures, and the final submission. RQ contributed to writing specific sections of the manuscript. RM-C and NB contributed to writing, editing, and discussing the manuscript. All authors read and approved the final manuscript.

## Conflict of Interest Statement

The authors declare that the research was conducted in the absence of any commercial or financial relationships that could be construed as a potential conflict of interest.
